# Molecular characterization, gene expression and dependence on thyroid hormones of two type I keratin genes (*sseKer1 *and *sseKer2*) in the flatfish Senegalese sole (*Solea senegalensis *Kaup)

**DOI:** 10.1186/1471-213X-7-118

**Published:** 2007-10-23

**Authors:** Carlos Infante, Manuel Manchado, Esther Asensio, José Pedro Cañavate

**Affiliations:** 1 IFAPA Centro El Toruño, Junta de Andalucía, Camino Tiro de pichón s/n, 11500 El Puerto de Santa María, Cádiz, Spain

## Abstract

**Background:**

Keratins make up the largest subgroup of intermediate filaments, and, in chordates, represent the most abundant proteins in epithelial cells. They have been associated with a wide range of functions in the cell, but little information is still available about their expression profile and regulation during flatfish metamorphosis. Senegalese sole (*Solea senegalensis*) is a commercially important flatfish in which no keratin gene has been described yet.

**Results:**

The development of large-scale genomics of Senegalese sole has facilitated the identification of two different type I keratin genes referred to as *sseKer1 *and *sseKer2*. Main characteristics and sequence identities with other fish and mammal keratins are described. Phylogenetic analyses grouped sseKer1 and sseKer2 in a significant clade with other teleost epidermal type I keratins, and have allowed for the identification of sseKer2 as a novel keratin. The expression profile of both genes was studied during larval development and in tissues using a real-time approach. *sseKer1 *and *sseKer2 *mRNA levels were significantly higher in skin than in other tissues examined. During metamorphosis, *sseKer1 *transcripts increased significantly at first stages, and reduced thereafter. In contrast, *sseKer2 *mRNA levels did not change during early metamorphosis although a significant drop at metamorphosis climax and late metamorphosis was also detected. To study the possible regulation of *sseKer *gene expressions by thyroid hormones (THs), larvae were exposed to the goitrogen thiourea (TU). TU-treated larvae exhibited higher *sseKer1 *and *sseKer2 *mRNA levels than untreated control at both 11 and 15 days after treatment. Moreover, addition of exogenous T4 hormone to TU-treated larvae restored or even reduced the steady-state levels with respect to the untreated control, demonstrating that expression of both genes is negatively regulated by THs.

**Conclusion:**

We have identified two keratin genes, referred to as *sseKer1 *and *sseKer2*, in Senegalese sole. Phylogenetic analyses revealed sseKer2 as a novel keratin. Although they exhibit different expression patterns during larval development, both of them are negatively regulated by THs. The co-regulation by THs could explain the reduction of both keratin transcripts after the metamorphosis climax, suggesting their role in the tissue remodelling processes that occur during metamorphosis.

## Background

In animal cells, the cytoskeleton is composed of three filament classes: actin microfilaments (6 nm diameter), microtubules (20 nm diameter), and intermediate filaments (IFs) so denoted for their size (8–12 nm diameter)[1,2]. The integrated network formed by all these filaments, in conjunction with associated proteins, is responsible for the mechanical integrity of the cell. In contrast to the evolutionarily conserved actin and tubulin proteins, IFs are highly divergent and exhibit cell typespecific expression patterns (reviewed in [3]). In human, up to 70 different IFs have been identified and grouped in five subfamilies, termed type I to V, with type I and type II including keratins [3,4].

Keratins are the major structural proteins in epithelial cells, and are encoded by a large multigene family that accounts for about 75% of all IF genes in chordates [5]. They act as a scaffold allowing for the maintenance of cellular architecture, and providing resistance to mechanical and non-mechanical stresses; although, other nonstructural functions such as cell signalling, protein targeting in polarized epithelia, cell proliferation, and in apoptosis have also been reported [5-11]. According to the biochemical properties of the encoded polypeptides, keratins are classified into two different types: type I and type II [1,9]. Type I keratins are acidic (pI = 4–6), and their molecular masses range from 40 to 64 kDa, whereas type II keratins are neutral to basic (pI = 6–8) and tend to be larger (52 to 68 kDa). Nevertheless, this designation of type I keratins as acidic or type II as basic is not supported in fish (see for example [12-14]). Unlike other IF proteins, keratins form obligatory heteropolymeric filaments consisting of equal numbers of type I and type II keratins [1,15]. All keratins share a common tripartite structure that includes a central highly α-helical "rod" domain, which features heptad repeats of hydrophobic residues, flanked by a "head" and a "tail" domain [1,9,16]. The heptad repeat pattern of the rod domain is a signature of a coiled-coil fold [17], which leads to an elongated architecture formed by a parallel coiled-coil dimer. Nevertheless, the heptad periodicity within the rod domain is interrupted in several places, resulting in four consecutive segments (or coils) of different size but identical number of amino acids (aa) among keratins (1A, 1B, 2A, and 2B), separated by three spacers or "linkers" (L1, L12, and L2) of highly variable length and primary structure [1,9,16].

According to their expression in epidermal keratinocytes as well as other stratified epithelia and simple epithelia, keratins are subdivided into two distinct types: "E" (from epidermal) and "S" (from simple epithelia) [18]. In teleosts, "S" keratins are also expressed in many mesenchymal cells, probably helping to resist osmotic stress [18]. In human, the classical "S" keratin 8 and keratin 18 [19] have true orthologs in tetrapods and teleosts. In contrast, teleost "E" keratins as well as other "S" keratins lack direct orthologs in other vertebrate classes, suggesting an independent evolution in bony fish [20-23]. This observation is also supported by sequence comparison and chromosomal distribution analyses of vertebrate keratin genes, which uncover a scattered distribution and an excess of type I keratins in teleosts in contrast to the two clusters and almost the same proportion of type I and type II keratin genes found in mammalian genomes [24-27].

Senegalese sole, *Solea senegalensis *(Pleuronectiformes: Soleidae), is a commercially important flatfish. During larval development, this species undergoes metamorphosis from 12 to 19 days after hatching (DAH). The process involves jaw and head restructuring, eye migration from the left to the right side, and a change from a symmetrical to an asymmetrical body shape [28]. These drastic morphological changes have been shown to be regulated by thyroid hormones (THs) in flatfish [29,30]. Currently, the regulation of keratin genes by THs has been demonstrated in other metamorphosing organisms such as *Xenopus laevis *and *Rana catesbeiana *[31-35]. In flatfish, only in Atlantic halibut (*Hippoglossus hippoglossus*) a keratin gene (*hhKer1*) has been recently characterized. This gene was shown to be down-regulated at the climax of metamorphosis, when T4 hormone reached the highest levels [36]. Nevertheless, it should be highlighted that dependence of keratin genes expression on THs during metamorphosis remains to be demonstrated in flatfish.

The development of large-scale genomics of Senegalese sole has allowed for the availability of a high number of EST sequences. In this study, we have identified and characterized two type I keratin genes (referred to as *sseKer1 *and *sseKer2*). The main sequence features are described. Phylogenetic analyses were carried out to identify putative orthologous sequences. Gene expression profiles during larval development and in tissues from juvenile soles were explored using real-time PCR. Additionally, thiourea (TU) and T4 treatments were carried out in order to elucidate the dependence of both *sseKer *gene expressions on THs.

## Results

### Molecular characterization and phylogeny of Senegalese sole keratins

Two *sseKer *genes were identified after EST analysis of a normalized cDNA library constructed from different larval stages (pre-, meta-, and post-metamorphosis), undifferentiated gonads, and six adult tissues (testis, ovary, stomach, intestine, liver, and brain). A total of 8 and 3 clones were identified as *sseKer1 *and *sseKer2*, respectively. Sequences of both *sseKer loci *were confirmed by direct sequencing of PCR products amplified from a pre-metamorphic library using all the possible combinations of keratin-specific primers (Table 1) with universal primers T3 and T7 (although modified by elongation of their 5'-end with 6 nucleotides using the pBK-CMV sequence as template). The PCR conditions were the same as used in real-time assays. This strategy also allowed us to rule out amplification of additional Senegalese sole keratin genes.

**Table 1:** Primers used for real-time gene expression analysis. The amplicon size generated by each primer pair is shown.

Target	Primers		Fragment size (bp)
		
	Primer pair name	Sequence	
*sseKer1*	SseK1•1	5'-AAATCCAGAACCGCTACGCCATGC-3' (F)	70
	SseK1•2	5'-AGCTGCTCCTCCATACCGCTCACC-3' (R)	
*sseKer2*	SseK2•1	5'-CGTCGCCTCCAAGAACCGCAGA-3' (F)	86
	SseK2•2	5'-CTGTGCTCACGGCCACCTCCTTA-3' (R)	
*GAPDH2*	SseGAPDH231•1	5'-AGCCACCGTGTCGCCGACCT-3' (F)	107
	SseGAPDH231•2	5'-AAAAGAGGAGATGGTGGGGGGTGGT-3' (R)	
*18S rRNA*	Sse18S•1	5'-GAATTGACGGAAGGGCACCACCAG-3' (F)	148
	Sse18S•2	5'-ACTAAGAACGGCCATGCACCACCAC-3' (R)	

*sseKer1 *encoded a transcript of 1441 nucleotides (nt) [DDBJ:AB301423] that contained a short 5'-untranslated region (42 nt) followed by an open reading frame (ORF) of 409 codons. The 3'-untranslated region was 172 nt long and included a canonical polyadenylation signal (AATAAA, 1422–1427) and a short oligo-A tail. For *sseKer2*, a cDNA sequence of 1299 nt was identified [DDBJ:AB301424]. The ORF comprised 404 codons, and it was preceded by a 14-nt 5'-untranslated region. The 73-nt long 3'-untranslated region contained a polyadenylation signal (AATAAA) at positions 1276–1281, and as *sseKer1*, included a short oligo-A tail.

The translated aa sequences of *sseKer1 *and *sseKer2 *showed that they consisted of 408 and 403 residues of molecular mass 44,188 and 44,332 Da, respectively. The predicted pI of sseKer1 and sseKer2 proteins was 4.91 and 5.11, respectively.

**Figure 1:**
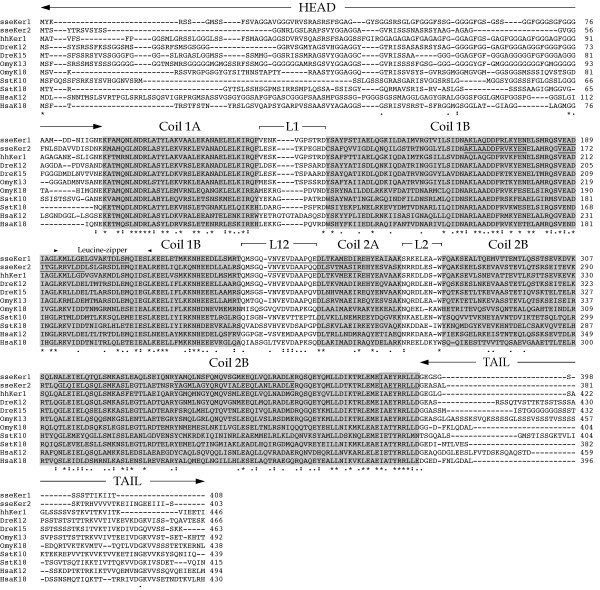
Comparison of the primary structure of sseKer1, sseKer2, and various others type I keratin proteins (see Table 2). The alignment was performed using MegAlign software. Both sseKer1 and sseKer2 exhibit the typical features of type I keratins. The helical subdomains (coil 1A, 1B, 2A, and 2B) are shaded in grey. "Head" and "tail" domains, as well as the non-helical linkers L1, L12, and L2, are also indicated. Asterisks denote identical amino acids; double dots and single dots indicate different degrees of amino acid conservation. Arrowheads show the location of the leucine-zipper found in sseKer1 and sseKer2. The IF signature motif found at the end of coil 2B is boxed. Finally, characteristic motifs present in type I keratins and detected in sseKer1 and sseKer2 are underlined.

A computational analysis of *sseKer1 *and *sseKer2 *using tBLASTx revealed that the best matches corresponded to teleost type I keratins in both cases. Both sseKer1 and sseKer2 contained a head domain (87 and 70 aa, respectively) rich in glycine and serine, and a short tail domain (16 and 28 aa, respectively). Features of IFs present in both sseKer1 and sseKer2 included a central rod domain with the coil 1A (35 residues: 88–122 in sseKer1, 71–105 in sseKer2), coil 1B (101 residues: 134–234 in sseKer1, 117–217 in sseKer2), coil 2A (19 residues: 251–269 in sseKer1, 234–252 in sseKer2), and coil 2B (115 residues: 278–392 in sseKer1, 261–375 in sseKer2), separated by the linker regions L1 (11 residues), L12 (16 residues) and L2 (8 residues), and the evolutionarily conserved IF signature motif IAEYRRLLD (Figure 1). In addition, five characteristic motifs of type I keratins were identified both in sseKer1 and sseKer2, two in each of coil 1B and 2B, and a fifth one spanning between L12 and coil 2A (Figure 1). Finally, a leucine-zipper motif L-x(6)-L-x(6)-L-x(6)-L was identified in coil 1B domain of both sseKer1 and sseKer2 at positions 193–214 and 176–197, respectively (Figure 1).

Sequence identities between sseKer1 and sseKer2, and with respect to type I keratins from other vertebrates (see Table 2), are depicted in Table 3. Identity between both Senegalese sole keratins was 54.1 (entire protein) and 63.6% (rod domain), respectively. With respect to other species, sseKer1 overall identities ranged between 36.5–73.8% and 42.3–81.6% for the entire protein and central rod domain, respectively. For sseKer2, amino acid identities varied between 39.0–67.5% and 45.7–77.0%, respectively. In all pairwise comparisons, sseKer1 and hhKer1 exhibited the highest identity values.

**Table 2:** Range of type I keratin sequences used in this study. The abbreviated name, description, accession number and species are indicated in each case.

Name	Description	Accession number	Species
AbaK10	Keratin 10	AJ493255	*Acipenser baeri*
AbaK11	Keratin 11	AJ493256	
AbaK12	Keratin 12	AJ493257	
AbaK13	Keratin 13	AJ493258	
AbaK14	Keratin 14	AJ493259	
AbaK15	Keratin 15	AJ493260	
AbaK18	Keratin 18	AJ493261	
CauK18	Keratin 18	L09744	*Carassius auratus*
CauK49	Keratin 49	L09743	
DreK11	Keratin 11	BC075874	*Danio rerio*
DreK12a	Keratin 12	NM_001003445	
DreK12b	Keratin 12	BC044144	
DreK13	Keratin 13	BC076059	
DreK14	Keratin 14	BC076485	
DreK15	Keratin 15	NM_213523	
DreK17a	Keratin 17	BC115108	
DreK17b	Keratin 17	BC092718	
DreK18a	Keratin 18	NM_178437	
DreK18a(K15)	Keratin 18	BC066541	
DreK18b	Keratin 18	AJ493269	
DreK18b(K16)	Keratin 18	BC078359	
DreK19	Keratin 19	BC097213	
DreK20	Keratin 20	AL645755	
DreK21	Keratin 21	BC115321	
DreK22	Keratin 22	BX248511	
DreKIc11d	Keratin type I c11d	NP_001002392	
DreKIc6	Keratin type I c6	NP_956862	
DreKtI	Keratin type I	NM_131108	
DreKtIel	Keratin type I enveloping layer	NP_571182	
hhKer1	Epidermal type I keratin	DQ364242	*Hippoglossus hippoglossus*
HsaK12	Keratin 12	D78367	*Homo sapiens*
HsaK18	Keratin 18	NM_002273	
HsaK20	Keratin 20	NM_019010	
HsahKtI	Type I hair keratin 1	Y16787	
LfuK10	Keratin 10	AJ308116	*Lampetra fluvialitis*
LfuK11	Keratin 11	AJ308117	
LfuK18	Keratin 18	AJ308118	
LocK10	Keratin 10	AM419763	*Lepisosteus oculatus*
LocK12	Keratin 12	AM419764	
LocK14	Keratin 14	AM419454	
MmuK10	Keratin 10	NM_010660	*Mus musculus*
MmuK18	Keratin 18	NM_010664	
OmyK10	Keratin 10	AJ272372	*Oncorhynchus mykiss*
OmyK11	Keratin 11	AJ272371	
OmyK12	Keratin 12	AJ427868	
OmyK13	Keratin 13	AJ427867	
OmyK18	Keratin 18	Y14289	
PseK10	Keratin 10	AM419450	*Polypterus senegalus*
PseK11	Keratin 11	AM419451	
PseK14a	Keratin 14	AM419452	
PseK14b	Keratin 14	AM419453	
PseK18a	Keratin 18	AM419448	
PseK18b	Keratin 18	AM419449	
PaeK12	Keratin 12	AJ785786	*Protopterus aethiopicus*
PaeK13	Keratin 13	AJ785787	
PaeK14	Keratin 14	AJ785788	
PaeK15	Keratin 15	AJ785789	
PaeK16	Keratin 16	AJ785790	
PaeK17	Keratin 17	AJ785791	
PaeK18	Keratin 18	AJ785792	
PaeK19	Keratin 19	AJ785793	
PaeK20	Keratin 20	AJ785799	
PaeK21	Keratin 21	AJ785785	
SstK10	Keratin 10	AJ623268	*Scyliorhinus stellaris*
SstK18	Keratin 18	Y14647	
TruKIb	Keratin type I b	SINFRUG00000155538	*Takifugu rubripes*
TruKIe	Keratin type I e	SINFRUG00000145680	
TruKIg	Keratin type I g	SINFRUG00000133679	
TruK18	Keratin 18	SINFRUG00000126133	
TniKtI	Keratin type I	GSTENP00036759001	*Tetraodon nigroviridis*
TniKIb	Keratin type I b	GSTENT00013816001	
TniKIe	Keratin type I e	GSTENT00026405001	
TniKIf	Keratin type I f	GSTENT00035830001	
XlaK18	Keratin 18	BC054993	*Xenopus laevis*
XK81	Gastrula stage epidermal type I cytokeratin	M11940	
XlaKtI	Type I cytokeratin	Y00968	

**Table 3:** Percentage of amino acid sequence identity among Senegalese sole keratins and several other type I keratins (see Table 2) as calculated using MegAlign. Values were determined considering the entire proteins (above diagonal) or the central rod domains (below diagonal).

	sseKer1	sseKer2	hhKer1	DreK12	DreK15	OmyK13	OmyK18	SstK10	SstK18	HsaK12	HsaK18
sseKer1	-	54.1	73.8	62.0	58.3	58.6	39.0	40.7	36.5	47.8	37.5
sseKer2	63.6	-	54.1	67.5	62.0	63.3	41.9	39.0	40.2	45.2	41.2
hhKer1	81.6	63.0	-	58.1	55.4	56.1	36.3	36.9	37.6	42.8	36.7
DreK12	69.8	77.0	69.8	-	68.3	67.4	44.7	40.5	44.1	47.9	40.9
DreK15	65.2	71.1	64.9	81.0	-	69.8	42.9	41.5	41.0	45.6	40.9
OmyK13	66.2	72.8	65.9	78.4	80.0	-	43.2	39.4	40.0	45.1	39.3
OmyK18	44.3	48.2	43.6	51.8	50.2	51.1	-	35.4	48.2	37.7	51.6
SstK10	44.4	45.7	42.1	47.7	48.0	46.1	42.8	-	40.0	37.4	36.5
SstK18	42.3	46.6	43.6	51.8	48.5	47.2	57.4	48.0	-	38.8	48.0
HsaK12	53.4	53.8	49.8	57.0	54.4	55.1	45.6	43.1	46.2	-	37.4
HsaK18	42.3	46.9	42.3	48.9	48.2	45.9	60.0	42.8	55.7	45.6	-

**Figure 2:**
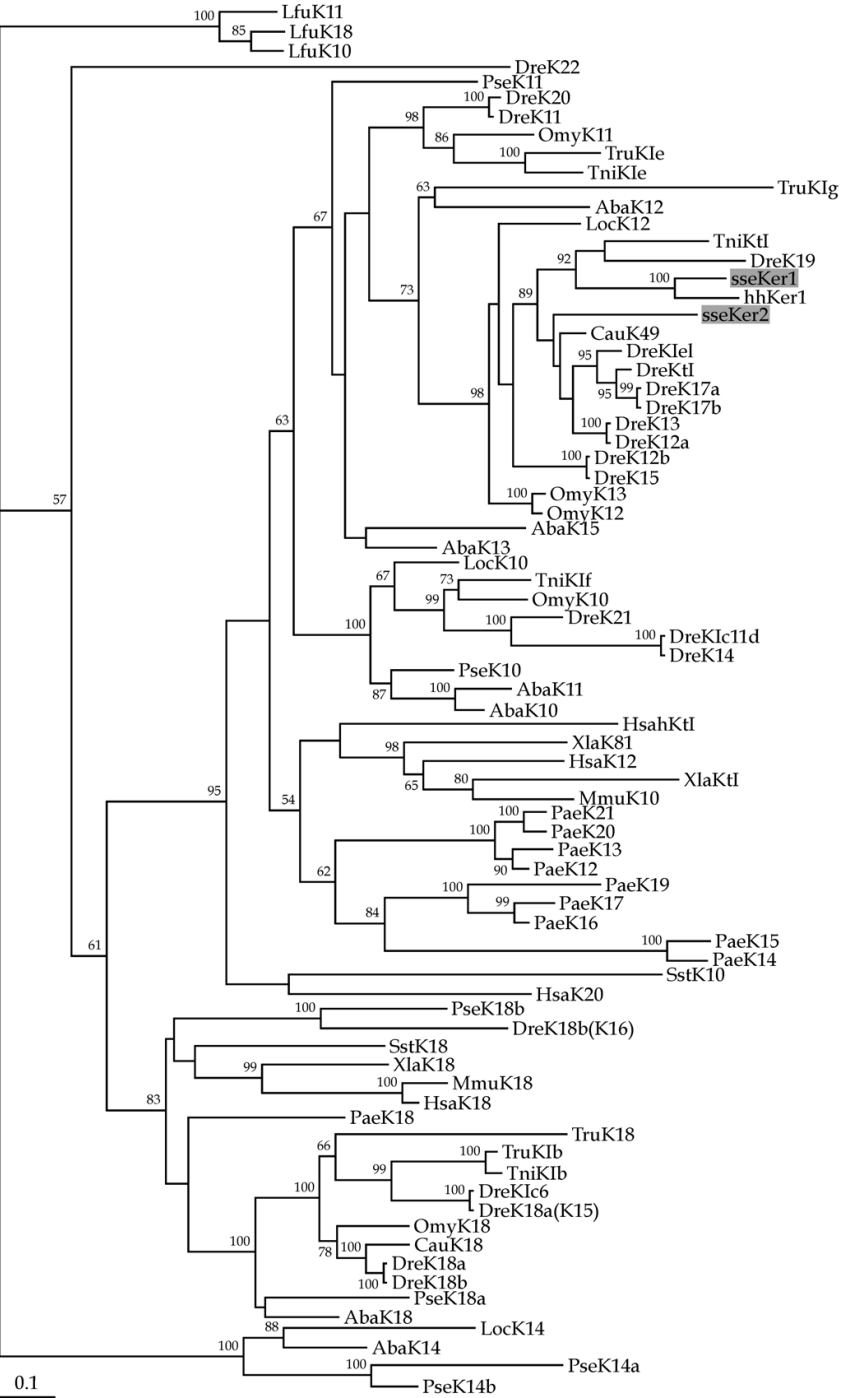
Phylogenetic relationships of sseKer1, sseKer2, and a wide range of vertebrate type I keratins (see Table 2) using the maximum likelihood method. River lamprey type I keratins were used as an outgroup to root tree. Only bootstrap values higher than 50% are indicated for each branch. The scale for branch length (0.1 substitutions/site) is shown below the tree. Both sseKer1 and sseKer2 appear closely related to epidermally expressed type I keratins from other teleosts. The Atlantic halibut keratin hhKer1 appears as the ortholog of sseKer1. In contrast, no counterpart for sseKer2 is found among the available teleost sequences included in the analysis.

**Figure 3:**
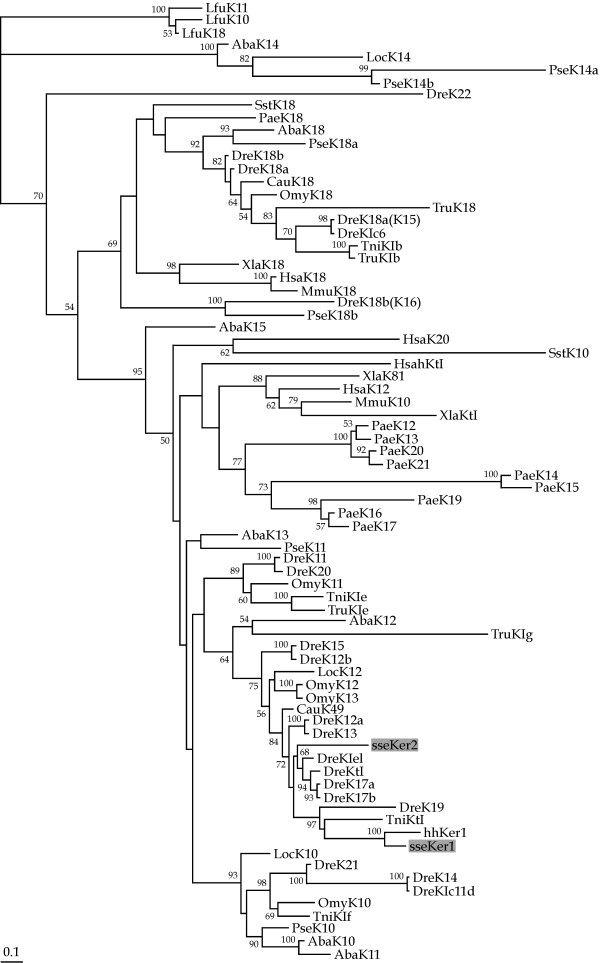
Phylogenetic relationships of sseKer1, sseKer2, and a wide range of vertebrate type I keratins (see Table 2) using the neighbor-joining method. River lamprey type I keratins were used as an outgroup to root tree. Only bootstrap values higher than 50% are indicated for each branch. The scale for branch length (0.1 substitutions/site) is shown below the tree. Results are largely congruent with those described for maximum likelihood method.

Maximum likelihood (ML) and neighbor-joining (NJ) phylogenetic trees were constructed from a multiple aa sequence alignment of the central rod domains corresponding to sseKer1, sseKer2, as well as a range of type I keratins (see Table 2). Largely congruent topological trees were generated using both methods (Figures 2 and 3). In agreement with previous reports, keratin 14 sequences only found in ancient ray-finned fish such as sturgeon (*Acipenser baeri*), bichir (*Polypterus senegalus*) and gar (*Lepisosteus oculatus*), appeared forming a separate and highly consistent clade (100% of bootstrap support) close to sequences of river lamprey (*Lampetra fluviatilis*) used as an outgroup [37]. Similarly, a consistent clustering of gnathostomian keratin 18 sequences was also observed both in ML and NJ trees. With regard to Senegalese sole keratins, they grouped together with other teleost epidermal keratins in a highly consistent clade (98 and 75% of bootstrap value in ML and NJ trees, respectively). Nevertheless, sseKer1 and sseKer2 clustered into different subclades. The former appeared closely related to hhKer1 in a highly supported branch (100% of bootstrap support both in ML and NJ trees). In contrast, sseKer2 appeared as a single ramification of another cluster that also included either CauK49 and several zebrafish (*Danio rerio*) keratins (ML tree; Figure 2), or only a reduced group of zebrafish keratins (NJ tree; Figure 3). It is important to highlight that a teleost sseKer2 counterpart was not found neither in ML nor in NJ trees.

### Expression levels of sseKer genes in tissues

**Figure 4:**
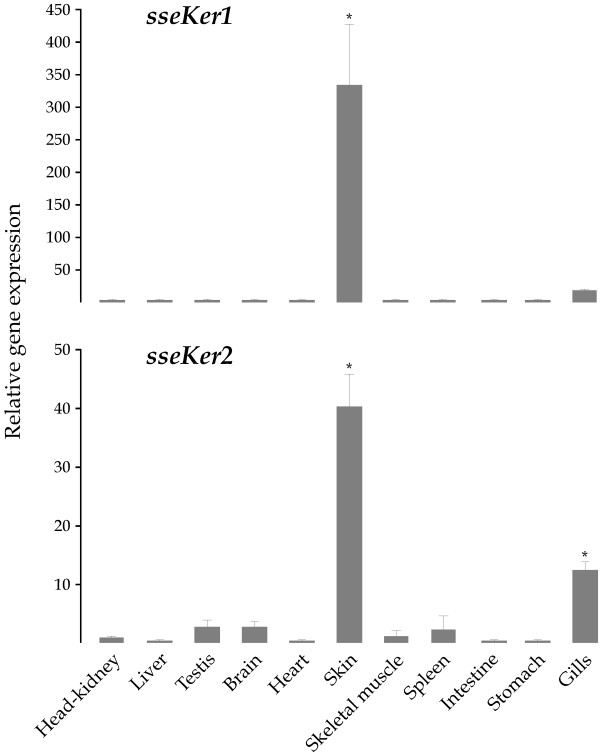
Relative *sseKer1 *and *sseKer2 *expression levels in tissues. Expression values were normalized to those of *18S rRNA*. Data were expressed as the mean fold change (mean ± SEM, n = 3) from the calibrator group (head-kidney). Values with asterisks are significantly different from head-kidney (*P *< 0.05). Both *sseKer1 *and *sseKer2 *exhibit significantly higher transcript levels in skin than in the rest of tissues.

Steady-state levels of both Senegalese sole keratin transcripts were quantitated in head-kidney, liver, testis, brain, heart, skin, skeletal muscle, spleen, intestine, stomach, and gills from juvenile soles (Figure 4). Relative gene expression levels were normalized by measuring *18S rRNA *and expressed relative to head-kidney. Both keratin genes exhibited a quite similar expression profile in tissues. *sseKer1 *transcripts were highly abundant in skin (17, 53, and 80-fold higher than gills, brain and intestine, respectively, and >200-fold higher than the remaining tissues; *P *< 0.001 in all cases). Likewise, *sseKer2 *was abundantly expressed in skin. However, this keratin gene also exhibited significantly higher mRNA levels in gills than in the remaining tissues (*P *< 0.001).

### Expression levels and regulation during larval development

**Figure 5:**
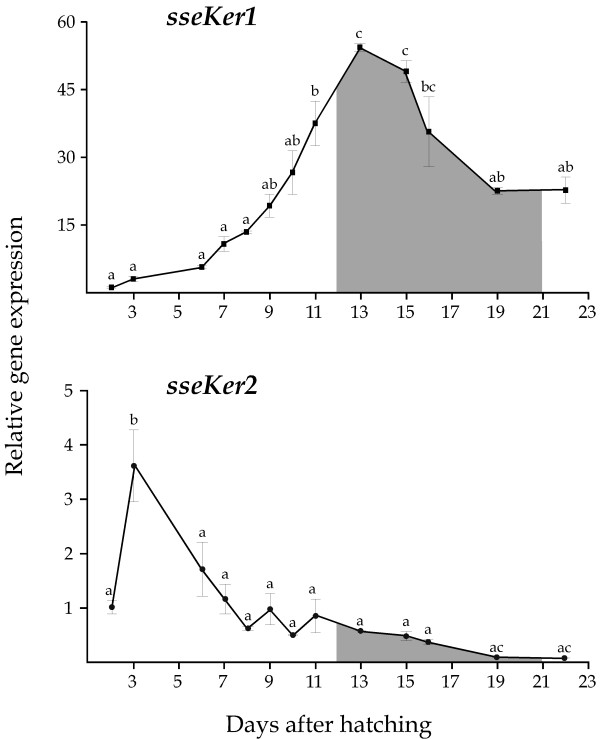
Relative *sseKer1 *and *sseKer2 *expression levels during larval development (from 2 to 22 DAH) in Senegalese sole. Expression values were normalized to those of *GAPDH2*. Data were expressed as the mean fold change (mean ± SEM, n = 3) from the calibrator group (2 DAH). Different letters denote days that are significantly different (*P *< 0.05), analyzed using ANOVA followed by a Tukey test. Double letters indicate intermediate states of statistical significance between the corresponding single letters. The interval for the metamorphic process is shaded. The expression profiles are quite different. Whereas *sseKer1 *reaches a peak at 13 DAH, the highest expression levels of *sseKer2 *are detected at 3 DAH, coinciding with external feeding.

Expression patterns of both Senegalese sole keratin genes during larval development (from 2 to 22 DAH) were also determined. Data were normalized to the housekeeping gene glyceraldehyde-3-phosphate dehydrogenase (*GAPDH2*; [DDBJ:AB291587])[38], and further expressed relative to 2 DAH. Both *sseKer1 *and *sseKer2 *transcripts were detected very early at 2 DAH (Figure 5). Nevertheless, they displayed different expression profiles during development. *sseKer1 *transcripts increased continuously reaching a peak at 13 DAH (54-fold, *P *< 0,001; S1 metamorphic stage). Thereafter, mRNA levels dropped progressively. In contrast, *sseKer2 *transcripts increased significantly at 3 DAH, coinciding with the external feeding (3.6-fold; *P *< 0.001). After that, expression levels did not vary significantly until the end of the metamorphic process, when a significant drop (*P *< 0.05) was detected (at 19 and 22 DAH).

**Figure 6:**
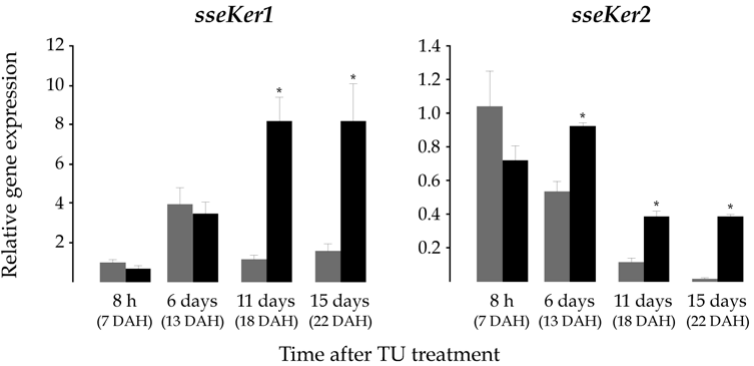
Relative *sseKer1 *and *sseKer2 *expression levels as determined by real-time quantitative PCR in the control (grey) and TU-treated (black) groups. Control and TU-treated samples were collected for RNA isolation at four different time periods (8 h, and 6, 11, and 15 days) after treatment 7 DAH. To facilitate comparisons, the number of DAH is indicated in each case. Expression values were normalized to those of *GAPDH2*. Data were expressed as the mean fold change (mean ± SEM, n = 3) from the calibrator (control 8 h). Values with asterisks are significantly different (*P *< 0.05 or lower) from the corresponding control group values. As a consequence of TU treatment, both *sseKer1 *and *sseKer2 *transcript levels were significantly higher in TU-treated larvae than in untreated larvae at 11 and 15 days after treatment.

**Figure 7:**
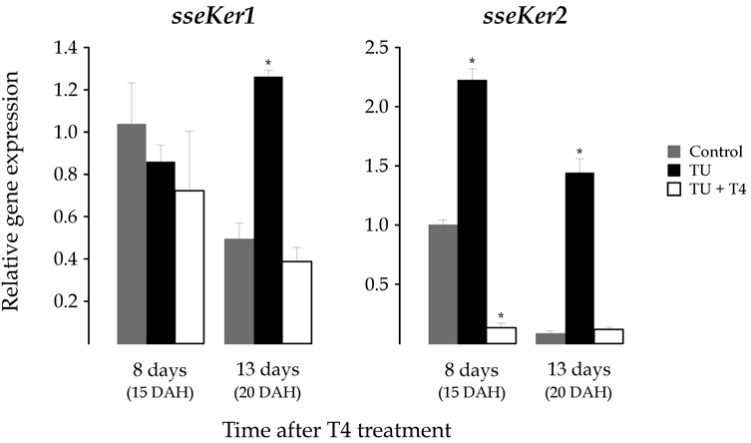
Relative *sseKer1 *and *sseKer2 *expression levels as determined by real-time quantitative PCR in the untreated control (grey), TU (black) and TU+T4 (white) treated groups. Larvae samples were collected for RNA isolation at 8 and 13 days after T4 treatment 7 DAH. To facilitate comparisons, the number of DAH is indicated in each case. Expression values were normalized to those of *GAPDH2*. Data were expressed as the mean fold change (mean ± SEM, n = 3) from the calibrator (untreated control day 8). Values with asterisks are significantly different (*P *< 0.05 or lower) from the corresponding control group values. Both *sseKer1 *and *sseKer2 *mRNA levels were similar or significantly lower in TU+T4-treated larvae than in untreated larvae.

To investigate the involvement of THs on the expression of both keratin genes, 7 DAH larvae were exposed to the goitrogen TU. As a consequence of the TU treatment, the metamorphic process was blocked at S1–S2 stages (early metamorphosis) as determined by the degree of eye migration [28]. No difference in survivability was observed with respect to untreated control (not shown). mRNA levels for both *sseKer *genes were quantified in whole larvae pools collected 8 h, and 6, 11, and 15 days after treatment (dat). Control larvae exhibited expression profiles similar to those described above (Figure 6). TU-treated larvae showed 7 and 5-fold higher *sseKer1 *mRNA levels (*P *< 0.05) than controls at both 11 and 15 dat, respectively. In contrast, no significant differences in gene expression were found at 6 dat (13 DAH larvae), suggesting that the rise of *sseKer1 *transcripts during metamorphosis (see Figure 5) is independent on THs. With regard to *sseKer2*, highly significant transcript levels were detected as a consequence of the TU treatment in larvae collected at 6 (1.7-fold; *P *< 0.05), 11 (3.3-fold; *P *< 0.01), and 15 (26.8-fold; *P *< 0.001) dat when compared to respective controls. At the sight of these results, we carried out a rescue assay in order to determine the ability of T4 to revert the TU effect on the expression of both keratin genes. For this purpose, 4 DAH larvae were exposed to TU to ensure the lack of endogenously synthethized T4 at day 7, when the exogenous T4 was supplied. mRNA levels of both *sseKer *genes were determined in larvae sampled 8 and 13 days after T4 treatment (datt). It is important to indicate that T4 released the metamorphosis blocking induced by TU. The expression patterns of untreated and TU-treated larvae were similar to those described above (Figures 5 and 6). In contrast, *sseKer1 *and *sseKer2 *steady-state transcript levels in TU+T4-treated larvae were similar or even significantly lower than those of untreated control (Figure 7).

## Discussion

We have obtained the complete cDNA sequence of two distinct Senegalese sole keratin genes, referred to as *sseKer1 *and *sseKer2*. Amino acid sequence analyses revealed that the subdomain organization was basically identical to that of mammalian and fish keratins in the central rod domain, detecting the four consecutive coils 1A, 1B, 2A, and 2B [3,9,12,21,39]. Moreover, both sseKer1 and sseKer2 presented the IF signature motif at the end of coil 2B, a feature that is common to all IFs, and possessed a predicted molecular mass and pI within the expected value for vertebrate type I keratins [1,19,21,23,33,34,39-41]. A leucine-zipper motif conserved among type I keratins from zebrafish, goldfish (*Carassius auratus*), rainbow trout (*Oncorhynchus mykiss*), *X. laevis*, and human was also identified at the end of coil 1B in both keratins [12]. All these results, and the identification of the five characteristic motifs that provide a signature for type I keratins, indicate that both *sseKer *genes actually encode for functional type I keratins.

Phylogenetic analyses give further support to the classification of sseKer1 and sseKer2 as "E" type I keratins. In fact, expression analysis of *sseKer *genes in tissues showed that the highest transcript levels were observed in skin. Both proteins were grouped together with other "E" type I keratins from teleosts [21,36] in highly consistent clades both in ML and NJ trees. At the sight of the phylogenetic trees, such a group of keratins does not seem to have a tetrapod counterpart. This result is not surprising if we take into account previous results showing that type I keratins from teleosts have diversified independently from those of tetrapods [4,21,25-27,37].

With the exception of *keratin 18 *gene, next to the type II *keratin 8 *gene, teleost type I genes are spread over many different positions in the genome. This may be the result of whole genome duplication and subsequent massive gene loss occurred in teleost fish lineage after its divergence from terrestrial vertebrates [42-44]. The high sequence identity and the close phylogenetic relatedness between sseKer1 and hhKer1 indicate that both genes could be considered as orthologous genes. However, this hypothesis is not supported by gene expression data. Whereas *hhKer1 *has an abundant expression in pre-metamorphic larvae [36], *sseKer1 *reaches the highest expression levels during metamorphosis (S1–S2 stages). In addition, *hhKer1 *is undetectable in adult skin [36]; in contrast, *sseKer1 *is abundantly expressed in skin from juvenile soles. So, if *hhKer1 *and *sseKer1 *are real (or not) orthologous genes, it is still a problem that is unresolved. With regard to sseKer2, no evident counterparts were found both in ML and NJ phylogenetic trees either in tetrapods or in teleosts, and thus sseKer2 can be considered as a novel type I keratin. *sseKer2 *could be the result of a type I gene duplication event and further retention in the Senegalese sole. Indeed, several examples of keratin duplications have been described in zebrafish, spotted green pufferfish (*Tetraodon nigroviridis*) and tiger puffer (*Takifugu rubripes*) [25]. We consider that orthologous genes for *sseKer2 *can be present in other teleosts, or, at least, in flatfish. Nevertheless, more available molecular data will be necessary to shed light on this issue.

Senegalese sole *sseKer1 *and *sseKer2 *showed quite different expression patterns during larval development. Whereas *sseKer1 *reached the highest transcript levels at S1–S2 metamorphic stages, *sseKer2 *was mostly expressed at initial developmental stages. However, both genes reduced their transcripts levels late in metamorphosis. Different expression profiles for epidermal keratin genes during metamorphic processes have also been described in anuran organisms. For example, a *X. laevis *51 kDa keratin coding gene was expressed in skin at an almost constant level during metamorphosis, while three 64 kDa keratin coding genes were up-regulated at a later larval developmental stage and further expressed highly in the adult skin [31,45]. Two other genes, XK70 and XK81, were expressed in the epidermis from embryos to larvae [46,47]. Moreover, type II *xlk *was expressed exclusively in the larval skin, and type I *xak-a*, *xak-b*, and *xak-c *genes in adult skin [34,35]. Finally, *xlk2*, another type I keratin gene, was exclusively expressed in the larval period from hatching to late metamorphic stages [41]. In developmental studies of *Rana catesbeiana *tadpoles, three epidermally expressed keratins have been further characterized: *rlk *and *rk8 *(type II), and *rak *(type I). During metamorphosis, no change in *rk8 *mRNA levels was detected whereas *rlk *and *rak *were down- and up-regulated, respectively [33,48]. Interestingly, the expression of many of these genes is differentially regulated by THs. Thus, 63 kDa keratin genes, *xak-a*, *xak-b*, *xak-c*, as well as *rak *are up-regulated by THs [31-35]. In contrast, *xlk *and *rlk *are negatively regulated [33-35].

THs play a key role in flatfish metamorphosis. The start of the metamorphic process has been associated with a surge of THs that increase their levels until the metamorphic climax, and reduce towards post-climax [29,30]. In this survey, the possible regulation of *sseKer1 *and *sseKer2 *by THs was studied using TU and combined TU+T4 treatments. TU is a blocking agent of THs synthesis that can reduce T4 levels by about 95% [49]. Exogenous treatments with TU as well as other types of thyroid inhibitors have proved useful to study the involvement of THs on metamorphosis in flatfish [29,49,50] and the transcriptional regulation of genes involved in the pituitary-thyroid axis [51,52]. TU-treated larvae exhibited significantly higher *sseKer1 *and *sseKer2 *transcript levels than controls at late metamorphosis. In contrast, addition of T4 reduced the amount of both *sseKer1 *and *sseKer2 *transcripts to similar or even significantly lower levels than those detected in controls. These results demonstrate that THs down-regulate *sseKer1 *and *sseKer2 *expression. The co-regulation of both keratins by THs, in spite of their distinct expression profiles during development, could explain the reduction of both keratin transcripts after the metamorphosis climax, suggesting their role in the tissue remodelling processes that occur during metamorphosis.

In fish, the absence of keratinization in adult skin seems to be a common phenomenon. Immunocytochemical and RT-PCR techniques have been unable to detect epidermal keratins typical of keratinizated tissues in the adult skin of several teleosts, including *C. auratus *and *H. hippoglossus *[36,53]. Thus, it has been proposed that scales and mucus present in adult skin of teleosts accomplish the same function as epidermal keratins and keratinizated skin in tetrapods, constituting a barrier against mechanical stress, parasites, pathogens, and loss of body fluids [36,54]. In fact, the presence of cystine bridges in the skin of adult Senegalese sole specimens has been related to the glycoprotein nature of the mucous secretion and not to a process of keratinization of the outer most layer of the epidermis [55]. Nevertheless, it is striking that among all tissues examined, both *sseKer1 *and *sseKer2 *exhibited the highest mRNA levels in skin. Thus, the possibility of both Senegalese sole keratin proteins playing a role in resistance to mechanical or osmotic stresses cannot be ruled out. This hypothesis is also supported by the abundance of the two *sseKer *transcripts in gills (mainly *sseKer2*), another mucus secreting tissue involved in osmotic regulation. Additionally, attractive hypotheses about other possible functions of sseKer1 and sseKer2 can be considered. For example, there are evidences supporting a role for keratin proteins in skin pigmentation both in human and mouse [8]. In the Senegalese sole, skin darkness has been observed in animals under stressing conditions [56]. As melanin granules have been found physically associated with IFs in skin pigment cells of fish [57], and there are accumulating reports of interactions of keratins (and other IFs) with microtubule-dependent motors [58], a putative role for Senegalese sole keratins in regulating the transport of melanin pigments is at least challenging. An alternative and exciting hypothesis for the possible involvement of sseKer1 or sseKer2 in skin pigmentation can be related to apoptotic processes. In this sense, there are findings that point to keratins as proteins involved in the regulation and execution of apoptosis [5], and such apoptotic processes seem to play a role in the change of body colour in fish by modifying the morphology and density of the skin pigment cells [59]. Moreover, it would be worthwhile to study the possible role of Senegalese sole keratins as regulators of protein synthesis and epithelial cell growth through interactions with components of the translational apparatus, as described for other keratins [8].

## Conclusion

In conclusion, in this study we describe the sequence and main features of the cDNAs encoding two type I keratins in the Senegalese sole, *sseKer1 *and *sseKer2*. Phylogenetic analyses revealed sseKer2 as a novel type I keratin. Both genes were highly expressed in skin and secondarily in gills. In contrast, they exhibited distinct expression patterns during larval development, with *sseKer1 *reaching the highest transcript levels during the first metamorphic stages and *sseKer2 *at first feeding. TU+T4 treatments demonstrated that *sseKer1 *and *sseKer2 *were negatively regulated by THs, suggesting their role in tissue remodelling that occurs during metamorphosis in flatfish. However, the specific function of each keratin during larval development and metamorphosis, as well as their regulation and interactions with other genes, are issues that need to be clarified in the future for the improvement of Senegalese sole aquaculture conditions.

## Methods

### Source of fish and experimental rearing conditions

All experimental sole larvae were obtained from fertilized eggs collected from breeding tanks, where breeders spawned naturally under environment conditions. Eggs were incubated at a density of 2000 eggs L^-1 ^in 300 L cylinder conical tanks with gentle aeration and a water exchange every two hours. Temperature and salinity during all experiments were 20°C and 38 ppt, respectively. Newly hatched larvae were transferred to a 400 L tank at an initial density from 45 to 50 larvae L^-1 ^with a 16L:8D photoperiod and a light intensity of 600–800 lux. Larvae were fed rotifers (*Brachionus plicatilis*) 3 DAH until 9 DAH. From 7 DAH enriched artemia metanauplii were fed until the end of the experiment. Pools of larvae from 2 to 22 DAH (n = 3) were collected, washed with DEPC water, frozen in liquid nitrogen, and stored at -80°C until analysis. Metamorphic stages (S0–S4) were classified according to [28]. These stages are as follows: S0 (pre-metamorphic), symmetric larvae with vertical swimming plane; S1, the left eye starts to migrate towards the dorsal position, until it touches the midline of the dorsal surface; S2, the migrating eye can be seen from the right-ocular side up to reaching the midline of the dorsal surface; S3 (metamorphic climax), the individuals change their swimming plane and the eye continues migrating within the ocular side; S4, eye translocation is completed and the orbital arch is clearly visible.

Chronic exposure to 30 ppm (394 μM) TU was achieved in 200 L round tanks. TU was added on 7 DAH, and water was kept stagnant for 24 h. After this exposure, 20% water was exchanged daily, with the subsequent addition of the eliminated TU. A second tank with the same characteristics was used to rear untreated control sole larvae. Larvae were initially stocked at a density of 100 individual L^-1^, and lights were kept off until the onset of external feeding 3 DAH. Fluorescent lamps supplied an illumination of 800 lux on the water surface, while a 16L:8D light cycle was used. Larvae were fed rotifers (*Brachionus plicatilis*) and 48 h *Artemia *metanauplii, according to the experimental design. The Haptophyceae *Isochrysis galbana *(T-ISO strain) was employed for the enrichment of both live preys, and was also added (2 mg dry weight L^-1 ^d^-1^) to larval tanks during rotifer use. Daily larval samples were removed for metamorphosis control. Pools of larvae (n = 3) were also collected, washed with DEPC water, frozen in liquid nitrogen, and stored at -80°C until analysis.

For the rescue experiments, culture conditions were the same as described above, but 30 ppm TU was added 4 DAH to ensure lack of endogenously synthethized T4 at the commencement of exogenous T4 treatment. At 7 DAH, untreated control larvae were transferred to a pair of 16 L cylinder conical tubes at an initital density of 45 larvae L^-1^. TU-treated larvae were transferred to 4 tubes, two of which were supplemented with 100 ppb (0.112 μM) T4. After this new exposure, 20% water was exchanged every two days, with the subsequent addition of the eliminated TU and TU+T4. Pools of larvae (n = 3) were collected 8 and 13 days after the commencement of T4 treatment, washed with DEPC water, frozen in liquid nitrogen, and stored at -80°C until analysis.

Juvenile Senegalese sole individuals (average weight = 169.60 ± 24.3 g; n = 3) were obtained from IFAPA Centro *El Toruño *facilities (El Puerto Santa María, Cádiz, Spain). They were sacrificed by immersion in tricaine methanesulfonate (MS-222) according to the guidelines on the care and use of fish in research, teaching and testing from the Canadian Council on Animal Care (2005). Head-kidney, liver, testis, brain, heart, skin, skeletal muscle, spleen, intestine, stomach, and gills were rapidly dissected, frozen in liquid nitrogen, and stored at -80°C until use.

### Identification of keratin cDNAs in Senegalese sole

Ten cDNA libraries were constructed from different larval stages and adult tissues of Senegalese sole using the ZAP Express^® ^cDNA Syntesis kit and Zap Express cDNA Gigapack^® ^III Gold Cloning kit (Stratagene) following the manufacturer's protocol. The libraries were pooled and normalized, and approximately 11,000 randomly selected clones were sequenced from the 3'-end (Cerdà et al., in preparation). Expressed sequence tags (ESTs) encoding keratins were identified after EST annotation. Sequences have been deposited with accession numbers [DDBJ:AB301423] and [DDBJ:AB301424] for *sseKer1 *and *sseKer2*, respectively.

Alignments of the predicted polypeptide sequences were carried out, and the sequence identities calculated by MegAlign program from the LASERGENE software suite. For this purpose, a range of type I keratin protein sequences were retrieved from databases (Table 2). ML and NJ phylogenetic analyses were carried out from a multiple amino acid sequence alignment of the rod domains corresponding to sseKer1, sseKer2 as well as a total of 76 additonal type I keratins (Table 2). The bestfit model of sequence evolution was determined to be JTT+I+G (-ln*L *= 21,898.95) using the ProtTest *v*1.3 [60], with a gamma distribution shape parameter (four rate categories) of 1.633, and a proportion of invariable sites of 0.081. These settings were also employed for the NJ analysis. In both cases, the PHYLIP package [61] was used as follows. A bootstrap analysis was performed using SEQBOOT (500 replicates for ML; 1000 replicates for NJ). Data were analyzed by ML using the software PHYML that generated 500 trees. The consensus phylogenetic tree was then obtained (CONSENSE). For NJ, data were analyzed with PROTDIST that generated 1000 distance matrices. The program NEIGHBOR was then employed to generate 1000 trees. Finally, the consensus phylogenetic tree was obtained using CONSENSE. Trees were drawn using the TreeViewX program *v*0.5.0. River lamprey type I keratins (Table 2) were used as and outgroup to root trees.

Presence of known domains in the predicted protein sequence of sseKer1 and sseKer2 was determined by scanning in the PROSITE database [62]. PSCAN application at the EMBOSS [63] was employed to identify type I keratin motifs.

### RNA isolation and gene expression analysis

Homogenization of juvenile tissues and larvae was carried out using Lysing Matrix D (Q-BioGene) for 40 s at speed setting 6 in the Fastprep FG120 instrument (Bio101). Total RNA was isolated from 50 mg of pooled larvae using the RNeasy Mini Kit (Qiagen). All RNA isolation procedures were performed in accordance with the manufacturer's protocol. In all cases, total RNA was treated twice with DNase I using the RNase-Free DNase kit (Qiagen) for 30 min in order to avoid amplification of contaminating genomic DNA. RNA sample quality was checked using Experion (Bio-Rad) and quantification was done spectrophotometrically. Total RNA (1 μg) from each sample was reverse-transcribed using the iScript™ cDNA Synthesis kit (Bio-Rad). Lack of genomic DNA contamination was confirmed by PCR amplification of RNA samples in the absence of cDNA synthesis.

Real-time analysis was carried out on an iCycler (Bio-Rad). Reactions were accomplished in a 25 μl volume containing cDNA generated from 10 ng of original RNA template, 300 nM each of specific forward (F) and reverse (R) primers (Table 1), and 12.5 μl of iQ™ SYBR Green Supermix (Bio-Rad). Matching oligonucleotide primers were designed using Oligo *v*6.89 software (Medprobe). Amplification of specific keratin encoding cDNA fragments was verified in previous assays by direct sequencing of PCR products obtained with the same reaction conditions employed in real-time PCR. The real-time amplification protocol used was as follows: initial 7 min denaturation and enzyme activation at 95°C, 40 cycles of 95°C for 15 s, and 70°C for 30 s. Each assay was done in duplicate. For normalization of cDNA loading, all samples were run in parallel using *GAPDH2 *or *18S rRNA *as housekeeping genes for larval development and tissues, respectively (Table 1). To estimate efficiencies, a standard curve was generated for each primer pair based on known quantities of cDNA (10-fold serial dilutions corresponding to cDNA transcribed from 100 to 0.01 ng of total RNA). All calibration curves exhibited correlation coefficients higher than 0.99, and the corresponding real-time PCR efficiencies were in the range 0.93–0.97. Relative mRNA expression was determined using the 2^-(ΔΔCt) ^method [64]. Results were expressed as mean ± SEM. Comparisons among groups were carried out with one-way analysis of variance (ANOVA), followed by a Tukey test for identification of the statistically distinct groups. Significance was accepted for *P *< 0.05.

## Abbreviations

DAH, days after hatching

dat, days after TU treatment

datt, days after T4 treatment

ML, maximum likelihood

NJ, neighbor-joining

SEM, standard error of the mean

THs, thyroid hormones

TU, thiourea

## Competing interests

The author(s) declare that there are no competing interests.

## Authors' contributions

EA performed the Senegalese sole cultures and hormone exposures, as well as larval and tissue samplings. CI and MM performed the relative quantitation assays and wrote the manuscript. JP-C designed the experiments, and participated in manuscript preparation. All authors read and approved the final manuscript.
